# Effectiveness of non-pharmacological falls prevention interventions for people with Multiple Sclerosis, Parkinson’s Disease and stroke: protocol for an umbrella review

**DOI:** 10.12688/hrbopenres.13023.2

**Published:** 2020-12-01

**Authors:** Nicola O'Malley, Amanda M. Clifford, Laura Comber, Susan Coote

**Affiliations:** 1 School of Allied Health, Faculty of Education and Health Sciences, University of Limerick, Limerick, Ireland; 2 Health Research Institute, University of Limerick, Limerick, Ireland; 3 Ageing Research Centre, Health Research Centre, University of Limerick, Limerick, Ireland; 4 Centre of Physical Activity for Health, Health Research Institute, University of Limerick, Limerick, Ireland

**Keywords:** Multiple Sclerosis, Parkinson’s Disease, Stroke, Falls, Intervention, Umbrella Review

## Abstract

**Background:** Falls are common among people with neurological diseases and have many negative physical, psychosocial and economic consequences. Implementation of single-diagnosis falls prevention interventions is currently problematic due to lack of participants and resources. Given the similarities in falls risk factors across stroke, Parkinson’s Disease (PD) and Multiple Sclerosis (MS), the development of an intervention designed for mixed neurological populations seems plausible and may provide a pragmatic solution to current implementation challenges. This umbrella review aims to summarise the totality of evidence regarding the effectiveness of non-pharmacological falls prevention interventions for people with MS, PD and stroke and identify the commonalities and differences between effective interventions for each disease to inform the development of an evidence-based intervention that can be tailored for people with mixed diagnoses.

**Methods:** This umbrella review will be conducted and reported in accordance with the Preferred Reporting Items for Systematic Reviews and Meta-Analyses (PRISMA) statement. 15 electronic databases and grey literature will be searched. Systematic reviews of randomised controlled trials and studies investigating the effects of non-pharmacological falls prevention interventions on falls outcomes among people with MS, PD and stroke will be included. Methodological quality of included reviews will be assessed using the Assessment of Multiple Systematic Reviews 2 tool. The Grading of Recommendations Assessments, Development and Evaluation framework will be used to rate the quality of evidence. A summary of evidence table and narrative synthesis will be utilised to clearly indicate the findings.

**Discussion:** This umbrella review presents a novel and timely approach to synthesise existing falls literature to identify effective non-pharmacological interventions for people with MS, PD and stroke. Of importance, a robust methodology will be used to explore the differences and similarities in effective interventions for individuals with these neurological conditions to facilitate the development of an intervention for these mixed neurological groups.

## Introduction

Neurological conditions are a leading cause of disability worldwide and, as a result, are associated with a large societal and economic burden ^1^. The global expenditure for disability secondary to neurological disorders has increased substantially over the past few decades, and is expected to increase further in the coming decades due to a rapid increase in population ageing ^1^. In Ireland, three of the most prevalent neurological conditions are Multiple Sclerosis (MS), Parkinson’s Disease (PD) and stroke ^2^. Fall rates are high among people with these neurological diseases and are often associated with many negative consequences. Therefore, the development of effective evidence-based falls prevention interventions for this cohort of individuals is a priority for research and service delivery. Up to 73% of stroke survivors experience a fall in the first year post-stroke ^3^ aand as many as 56% of people with MS fall in any given three-month period ^4^. Similarly, 59% of people with PD report having at least one fall over a six-month period ^5^. Physical injuries are a common consequence of a fall among people with neurological diseases with between 11–17% of falls resulting in injury ^6– 8^ but notably, this figure has been as high as 72% among stroke survivors ^9^. Falls also have a number of psychosocial impacts including fear of falling and reduced self-efficacy ^10, 11^, leading to decreased independence, reduced social participation and diminished health-related quality of life ^11, 12^. Additionally, falls result in increased acute healthcare utilisation, higher home-care needs and/or greater institutional care needs ^7– 9, 13^. This high rate of falls and associated physical, social and economic consequences highlights the need for an effective falls prevention intervention.

Recently there has been an increase in the number of interventions developed and evaluated for falls prevention among individuals with one specific neurological disease. This condition-specific approach to intervention is reflected in clinical practice where provision of services is typically disease-specific ^14^. However, implementation of these interventions in the community is a challenge as finding sufficient numbers and resources to run single-diagnosis groups is problematic for clinicians ^15^. The National Strategy & Policy for the Provision of Neuro-Rehabilitation Services in Ireland has demonstrated the current deficits in services available to people with neurological diseases and the associated negative consequences at both the individual and system level ^14^. This implementation strategy highlights the need for high-quality, person-centred care and timely access to services for people with neurological diseases to optimise outcomes ^14^. One potential solution is the development of interventions that can be implemented with mixed neurological conditions rather than disease-specific groups. Little is currently known about the feasibility or effectiveness of adopting this mixed population approach to falls rehabilitation. A scoping literature search revealed only one study examining the effect of a falls prevention intervention for people with MS, PD and stroke. This study found an educational programme supplemented with home exercises did not reduce falls among participants ^16^. However, the sparsity of evidence in this field means that further research is required before firm conclusions regarding the effectiveness of falls prevention interventions for these mixed-diagnosis groups can be drawn.

While the pathophysiology of stroke, PD and MS differs ^17– 20^, there are similarities in the presenting impairments and falls risk factors across the three diagnoses. People with MS, PD and stroke share a number of physiological and psychosocial falls risk factors including impaired mobility, reduced balance, cognitive deficits, decreased strength, depression, fear of falling and reduced ability to perform activities of daily living ^21– 27^, in addition to behavioural and environmental falls risk factors. Physiotherapists specialising in neurology or working in primary care usually manage individuals with each of these mixed neurological diseases in their practices and so, given the commonalities in these modifiable falls risk factors, it is likely that the subsequent goals of rehabilitation and treatment approaches used across diagnoses are also similar to reduce falls. This similarity in treatment approaches is reflected in research, where exercise with the aim of improving strength and balance appears to be the main component of many falls prevention interventions for people with PD, MS and stroke ^28– 30^. Therefore, it is hypothesised that programmes for mixed neurological groups comprising of people with MS, PD and stroke are feasible. It is acknowledged that there will be some variation in clinical presentation between people with MS, PD and stroke; however, tailoring of a programme to an individual’s unique presentation is required for all interventions, independent of diagnosis. Many falls prevention interventions contain core elements underpinning the content and delivery of the programme, in addition to person-specific, individualised components; thus it is anticipated that this model could also be used to develop a programme for people with MS, PD and stroke that can be adapted based on individual falls risk assessments. A mixed population approach to the development and provision of interventions has the potential to increase the number of eligible participants, reduce strain on healthcare resources and increase the number of services available to community-dwelling individuals living with PD, MS and stroke, thereby meeting the rehabilitation needs of these individuals while simultaneously negating the negative effects associated with insufficient service provision. Therefore, the development of an intervention for individuals with these mixed neurological diseases is timely to address the current implementation and service provision challenges in the community.

Following the Medical Research Council’s Framework, the first step in developing a complex intervention is the collation of the existing evidence-base ^31^. Therefore, to develop an intervention that is implementable across diagnoses, it is necessary to first identify what elements of existing programmes are effective for each condition. A recent umbrella review was the first of its kind to investigate the effectiveness of exercise-only interventions at reducing falls for people with neurological diseases, but a limitation of that review is the consideration of exercise interventions only ^32^. This study concluded that exercise interventions were effective at reducing falls for people with PD, but insufficient evidence existed to support their effectiveness for people with stroke or MS ^32^. Falls are widely accepted as having multifactorial causes, with a combination of physiological, behavioural, environmental and socioeconomic factors believed to influence falls risk ^33, 34^. Given the broad range of falls risk factors among people with neurological diseases ^6, 22, 23, 27, 35, 36^, a multimodal approach to falls prevention that targets a number of these risk factors simultaneously appears intuitive and has been suggested to address modifiable falls risk factors ^27^. Therefore, to develop an intervention that addresses the multifactorial nature of falls, there is a need to review the effectiveness of all non-pharmacological interventions across stroke, PD and MS. This umbrella review is novel in that it will use a robust methodology to assess the effectiveness of all non-pharmacological interventions, taking into consideration the multifactorial nature of falls. Additionally, this umbrella review will be the first of its kind to compare and contrast the effectiveness of interventions across diseases to facilitate the development of mixed neurological group interventions. These comparisons will consist of further sub-analyses to account for the heterogeneity both within and across the diagnoses of MS, PD and stroke, with respect to disease duration, functional ability and disease subtype.

The objectives of this umbrella review are:

1. To summarise the totality of evidence regarding the effectiveness of non-pharmacological falls prevention interventions for people with MS, PD and stroke.
2. To identify commonalities and differences between interventions that are effective at reducing falls for people with MS, PD and stroke to inform the development of an intervention for these mixed neurological groups.

## Methods

### Protocol and registration

An umbrella review will be conducted to identify systematic reviews (with or without meta-analysis) of studies investigating the effectiveness of non-pharmacological interventions to prevent falls among people with neurological diseases. In line with recommendations to improve transparency and reduce bias, this protocol was developed to outline the key objectives of this umbrella review and what methodology will be employed ^37^. This protocol was designed using the guidance of the relevant items of the Preferred Reporting Items for Systematic Reviews and Meta-Analysis Protocols (PRISMA-P) statement ^38^, with reference to the Joanna Briggs Institute (JBI) Reviewer’s Manual ^39^ and the PRISMA guidelines ^40, 41^. The PRISMA-P was developed to facilitate the design of protocols for systematic reviews, however, the relevant sections of the checklist will be used for this protocol in the absence of specific guidelines for the conduction and reporting of umbrella reviews ^42^. The protocol was registered with the International Prospective Register of Systematic Reviews, PROSPERO, CRD42020175409.

### Search strategy

The following electronic databases will be searched by one reviewer (NO’M) to identify potentially relevant reviews: The Cochrane Database of Systematic Reviews, Joanna Briggs Institute Database of Systematic Reviews and Implementation Reports, Database of Abstracts of Reviews of Effects, PubMed, Embase, Ebsco (Academic Search Complete, AMED, Biomedical Reference Collection, CINAHL, Medline, PsycInfo, SPORTDiscus), Epistemonikos, PEDro and the PROSPERO register. The authors developed a comprehensive search strategy to identify papers relevant to the primary aims of the overview. To illustrate, the full electronic database search string for the CINAHL database is detailed in Box 1. In addition, reference lists of included reviews will be hand-searched to identify other potentially relevant reviews. In line with best practice guidelines for the conduction of umbrella reviews, our comprehensive search will also encompass a search for grey literature ^43^.

**Box 1. Search Strategy for CINAHL**

**S1:** TI (falls OR fall* OR “accidental fall”) OR AB (falls OR fall* OR “accidental fall”)

**S2:** TI (stroke OR CVA OR cerebrovascular OR apoplexy OR vascular OR MS OR “multiple sclerosis” OR demyelin* OR PD OR “parkinson’s disease” OR “parkinson disease” OR parkinson* OR neurol*) OR AB (stroke OR CVA OR cerebrovascular OR apoplexy OR vascular OR MS OR “multiple sclerosis” OR demyelin* OR PD OR “parkinson’s disease” OR “parkinson disease” OR parkinson* OR neurol*)

**S3:** (TI stroke OR CVA OR cerebrovascular OR apoplexy OR vascular OR MS OR “multiple sclerosis” OR demyelin* OR PD OR “parkinson’s disease” OR “parkinson disease” OR parkinson* OR neurol* OR AB stroke OR CVA OR cerebrovascular OR apoplexy OR vascular OR MS OR “multiple sclerosis” OR demyelin* OR PD OR “parkinson’s disease” OR “parkinson disease” OR parkinson* OR neurol*) AND (S1 AND S2)

**S4:** TI (intervention OR prevention OR rehabilitation OR treatment OR therap*) OR AB (intervention OR prevention OR rehabilitation OR treatment OR therap*)

**S5:** (TI intervention OR prevention OR rehabilitation OR treatment OR therap* OR AB intervention OR prevention OR rehabilitation OR treatment OR therap*) AND (S3 AND S4)

**S6:** TI (systematic OR review OR “meta-analysis”) OR AB (systematic OR review OR “meta-analysis”)

**S7:** (TI systematic OR review OR “meta-analysis” OR AB systematic OR review OR “meta-analysis”) AND (S5 AND S6)

### Inclusion and exclusion criteria

This umbrella review will include quantitative systematic reviews (with or without meta-analysis), mixed-methods systematic reviews (quantitative elements only will be included) or pooled analyses and research syntheses investigating the effectiveness of falls prevention interventions for people with MS, PD and stroke. This umbrella review will include only research syntheses published in the English language due to resources. No restriction will be placed on date of publication. If a review is an update of a previous review, the most recent update will be included and the older versions will be excluded. An update of a systematic review has changes pertaining to new data, new methods, or new analyses, however, the research question, objectives and inclusion criteria remain similar ^44^. This updated review may be conducted by the same authors as the previous review or the research team may comprise of new authors. In the case of new authors updating an existing review, they must clearly state that their review is an update and acknowledge the work of the authors on the previous edition ^44^.

Potentially relevant papers will be assessed for inclusion as a systematic review by two independent reviewers (NO’M and AC/SC) using the JBI Critical Appraisal Checklist for Systematic Reviews and Research Syntheses ^39^. Any disagreements between reviewers will be resolved through discussion or by a third reviewer (AC/SC) until consensus is achieved. Any review that receives a ‘No’ response to any of the following will not be included ^45, 46^:

- Were the inclusion criteria appropriate for the review question? (Item 2)
- Was the search strategy appropriate? (Item 3)
- Were the sources and resources used to search for the studies appropriate? (Item 4)
- Were the criteria for appraising studies appropriate? (Item 5)
- Were the methods used to combine studies appropriate? (Item 8)

Upon completion of this appraisal, literature reviews that do not include these key features of accepted systematic review methodology, outlined by JBI ^47^, will be excluded from this umbrella review. If necessary, the authors of the reviews will be contacted to clarify any unclear or missing details before the review is excluded.

The inclusion criteria based on population, intervention, comparison, outcome and study design (PICOS) are outlined in Table 1.

**Table 1.** Summary of inclusion criteria for systematic reviews of falls prevention interventions.

Study characteristic	Inclusion criteria
Population	Adult participants (>18 years) with Parkinson’s Disease according to a confirmed diagnostic criterion at any stage of the Hoehn and Yahr Scale Adult participants with Multiple Sclerosis (MS) according to a confirmed diagnostic criterion with any subtype of MS such as relapsing remitting, primary progressive and secondary progressive Adult participants post-stroke, both ischaemic and haemorrhagic, in the hyperacute, acute, early subacute, late subacute or chronic phases following stroke Studies with a combination of the above populations Studies in which data regarding the above populations can be extracted There will be no exclusion based on gender, disease duration or functional ability For the purposes of this umbrella review, there will be no exclusion based on the presence of co-morbidities, however, it is likely that restrictions based on the presence of co-morbidities will be feature of included systematic reviews
Intervention	Non-pharmacological and non-surgical falls prevention interventions Any intervention in which a primary or secondary outcome was to reduce falls will be considered a falls prevention intervention Given the multifactorial nature of falls, and for inclusivity, there will be no exclusion based on intervention content, intervention duration, intervention setting or mode of intervention delivery
Comparison	In instances where controlled trials are included in the systematic reviews the following will be considered acceptable comparators: usual or enhanced care, or waitlist control
Outcomes	The primary outcomes of interest in this umbrella review are any falls outcomes measured as a primary or secondary outcome in included systematic reviews (for the purposes of this umbrella review, the occurrence of a fall event had to be recorded in order to be considered a falls outcome) This includes, but is not limited to, total number of falls, falls rate, number of fallers, number of recurrent fallers or injurious falls Given that there is currently no consensus regarding what constitutes a fall, in addition to the variation in fall definitions present in the literature, a pre-determined definition for a fall event will not be used in this umbrella review Instead, all systematic reviews will be included regardless of their definition of a fall, but these definitions will be extracted and presented to help readers contextualise the resultsSecondary outcomes of interest for this umbrella review are those relating to the effectiveness and implementability of interventions including, but not limited to, strength, mobility, fatigue, participation, balance, falls risk, adverse events or attrition rates Secondary outcomes will only be extracted in instances where falls were measured as a primary outcome and where it is possible to extract this data for our populations and interventions of interest
Study design	Systematic reviews of randomised controlled trials (RCTs) and quasi-RCTs Systematic reviews of RCTs and all other study designs investigating falls prevention interventions

### Study selection

The papers yielded from the search of each individual electronic database will be exported to the master reference management library Rayyan, where duplicate papers will then be removed. The titles and abstracts will be screened by two reviewers (NO’M and AC/SC) against the eligibility criteria for any obviously irrelevant papers. Following this, the full text of potentially relevant reviews will be screened by two independent reviewers (NO’M and AC/SC) to confirm inclusion in the final overview of reviews. Any discrepancies between reviewers will be resolved through a discussion or by a third reviewer (AC/SC) until consensus is achieved. A PRISMA flow diagram of the included studies will be completed.

### Data extraction

Data will be extracted by one reviewer (NO’M) using a standardised data extraction form. A second reviewer (LC) will then check the form to ensure that the extracted data are accurate. Disagreements regarding data extraction will be resolved through discussion or by consulting a third reviewer (AC/SC) until consensus is achieved. The data extraction form will include the following:

1. Citation details of included review
2. Objectives of included review
3. Type of review
4. Participant characteristics
5. Setting and context of the review
6. Number of databases searched
7. Date range over which database searching was conducted
8. Date range over which studies included in the review that inform each outcome of interest were published
9. Number of studies, types of studies and country of origin of studies included in each review
10. Instrument used to critically appraise the primary studies and their quality rating
11. Primary falls outcomes and secondary outcomes of interest reported in reviews
12. Methods employed to synthesise the evidence
13. Any comments or notes that the authors have regarding the included review

### Methodological quality assessment

The methodological quality of included reviews will be assessed by two independent reviewers (NO’M) using the Assessment of Multiple Systematic Reviews 2 (AMSTAR 2) tool ^48^. The AMSTAR 2 is a 16-item checklist utilised to assess the quality of systematic reviews that include randomised or non-randomised studies of healthcare interventions. Reviewers score each domain with ‘yes’ or ‘no’, or in some domains there is a third option of ‘partial yes’. The overall score of the AMSTAR 2 will be used to rate the quality of each included review investigating the effectiveness of falls prevention interventions as high, moderate, low or critically low ^48^. In line with recommendations ^48^, the following will be considered critical domains for the AMSTAR 2:

- Protocol registered before commencement of the review (item 2)
- Adequacy of the literature search (item 4)
- Justification for excluding individual studies (item 7)
- Risk of bias from individual studies being included in the review (item 9)
- Appropriateness of meta-analytical methods (item 11)
- Consideration of risk of bias when interpreting the results of the review (item 13)
- Assessment of presence and likely impact of publication bias (item 15)

The overall confidence in the results of a systematic review will be considered high if it has no or one non-critical weakness, moderate if more than one non-critical weakness is present, low if there is one critical flaw with or without non-critical weaknesses present, and critically low if there is more than one critical flaw with or without non-critical weaknesses ^48^.

It has been suggested that the use of PRISMA in conjunction with a comprehensive, validated critical appraisal tool facilitates judgement not only of the methodological quality of the included reviews but also the general quality of reporting ^49^. Consequently, the full text of all included reviews will be cross-checked against the PRISMA reporting guidelines checklist ^40, 41^.

### Quality of evidence

The Grading of Recommendations Assessments, Development and Evaluation (GRADE) framework was designed to provide guidance for rating the quality of evidence and grading the strength of recommendations in healthcare ^50^. This approach is primarily used to assess the quality of evidence in systematic reviews, but has been also applied to umbrella reviews in the absence of a more specific framework. The GRADE approach will be used to assess the quality of the evidence relating to the following outcomes included in RCTs in systematic reviews:

1. Total number of falls – the number of falls recorded by participants throughout the study period
2. Falls rate – the number of falls per person per specific period of time, e.g. falls per person per year
3. Number of fallers - the proportion of participants classified as ‘fallers’ based on the criteria outlined by the researchers e.g. an individual who has one or more falls during the follow-up period (Note: it is anticipated that the classification for a ‘faller’ will differ between reviews ^51^, if this is the case the reviewers will present these differences and discuss the potential impact on the results).

### Overlap of primary studies

Overlap of primary studies is a challenge unique to umbrella reviews. Presently, there is an absence of guidance on how best to deal with this phenomenon ^52^. In the presence of complete overlap between reviews, the highest quality review, as determined by the AMSTAR 2, will be included in data synthesis and analysis. In cases, where there is complete overlap and the reviews receive the same rating using the AMSTAR 2, then the most recently published review will be included. In the presence of partial overlap, all reviews will be included but the authors will note the degree of duplication and discuss its implications on the findings of this umbrella review.

### Discordance between reviews

There are a number of reasons for discordant reviews and the conduction of umbrella reviews allows researchers to address the issue of discordance and identify its cause ^49^. In the event of discordant reviews in our overview, the algorithm designed by Jadad *et al*. (1997) will be utilised to resolve issues of discordance ^53^.

### Data synthesis and analysis

This umbrella review will provide a summary of evidence table that will name the intervention, outline the included research synthesis and provide a clear indication of the results. We will endeavour to have a standardised approach to our results by converting the different estimates of effect that we extract to one common effect measure. However, these analyses will be contingent on several factors including access to raw data, whether the authors of the included systematic reviews performed meta-analyses and if the included systematic reviews have analysed the same falls outcomes. Given the anticipated heterogeneity in populations, outcomes and analyses, the findings of included reviews will likely be primarily summarised using a narrative synthesis with the quantitative tabulation of results as appropriate. The primary analyses for this umbrella review will be centred on type of neurological condition and type of intervention. Following this, cross-comparison of similarities and differences in the effect of different interventions between the three conditions will be performed. If the relevant data are presented in the included reviews, sub-analyses based on intervention dose, disease duration, functional ability and disease subtype will be completed. Where possible, the sensitivity of the review findings will be considered in the context of its methodological quality, as determined by the AMSTAR 2, to examine the effects of synthesising reviews of varying quality. In the first instance, analyses will be completed using systematic reviews of any methodological quality that include all study designs, followed by a second analysis using only systematic reviews with highest quality evidence (RCTs only). Comparisons between the two analyses will then be presented and discussed.

### Dissemination

The findings of this umbrella review will be disseminated through the publication of peer-reviewed manuscripts. Additionally, findings will be presented at both national and international conferences.

## Study status

The authors have commenced searches for this umbrella review.

## Discussion

This umbrella review will use a robust methodology to present evidence regarding the effectiveness of non-pharmacological falls prevention interventions on falls outcomes among individuals with MS, PD and stroke. The development of falls prevention interventions for groups with mixed neurological diseases may improve the implementability of programmes in the community. Given the sparsity of studies investigating the effectiveness of interventions across several neurological diseases, an umbrella review presents a novel approach to synthesise existing falls literature to identify similarities or differences in effective interventions for people with stroke, MS and PD to facilitate the development of a mixed diagnoses intervention. This umbrella review will be the first of its kind to investigate the effectiveness of all non-pharmacological falls prevention interventions across several neurological diseases.

## Data availability

### Underlying data

No data are associated with this article.

### Reporting guidelines

Figshare: PRISMA-P checklist for ‘Effectiveness of non-pharmacological falls prevention interventions for people with Multiple Sclerosis, Parkinson’s Disease and stroke: Protocol for an umbrella review’, https://doi.org/10.6084/m9.figshare.12063657.v1 ^54^.

Data are available under the terms of the Creative Commons Attribution 4.0 International license (CC-BY 4.0).
