# Supplementary material for: Effectiveness of non-pharmacological falls prevention interventions for people with Multiple Sclerosis, Parkinson’s Disease and stroke: protocol for an umbrella review
Source: HRB Open Res. 2020 Dec 1;3:17. Originally published 2020 Apr 24. [Version 2] doi: 10.12688/hrbopenres.13023.2 (PMC7745191; doi:10.12688/hrbopenres.13023.2)
Supplement: Peer review history

# Peer review: Reviewer response for version 2

**DOI:** 10.21956/hrbopenres.14327.r28483

**Published:** 2020-12-09

**Authors:** Ylva Nilsagård

**Affiliations:** 1 Health Care Management, Faculty of Medicine and Health, Örebro University, Örebro, Sweden

Is the study design appropriate for the research question?

Yes

Is the rationale for, and objectives of, the study clearly described?

Partly

Are sufficient details of the methods provided to allow replication by others?

Yes

Are the datasets clearly presented in a useable and accessible format?

Not applicable

Reviewer Expertise:

Falls and balance training in people with MS

I confirm that I have read this submission and believe that I have an appropriate level of expertise to confirm that it is of an acceptable scientific standard.

# Peer review: Reviewer response for version 2

**DOI:** 10.21956/hrbopenres.14327.r28484

**Published:** 2020-12-08

**Authors:** Feng Yang, Caroline Simpkins

**Affiliations:** 1 Department of Kinesiology and Health, Georgia State University, Atlanta, GA, USA

The authors have adequately addressed all of our concerns.

Is the study design appropriate for the research question?

Yes

Is the rationale for, and objectives of, the study clearly described?

Yes

Are sufficient details of the methods provided to allow replication by others?

Partly

Are the datasets clearly presented in a useable and accessible format?

Not applicable

Reviewer Expertise:

Fall prevention and neurorehabilitation.

We confirm that we have read this submission and believe that we have an appropriate level of expertise to confirm that it is of an acceptable scientific standard.

# Peer review: Reviewer response for version 2

**DOI:** 10.21956/hrbopenres.14327.r28485

**Published:** 2020-12-08

**Authors:** Vicki L Gray

**Affiliations:** 1 Department of Physical Therapy and Rehabilitation Science, University of Maryland School of Medicine, Baltimore, MD, USA

The authors have adequately addressed all of my concerns.

Is the study design appropriate for the research question?

Yes

Is the rationale for, and objectives of, the study clearly described?

Partly

Are sufficient details of the methods provided to allow replication by others?

Partly

Are the datasets clearly presented in a useable and accessible format?

Not applicable

Reviewer Expertise:

Balance and falls in stroke.

I confirm that I have read this submission and believe that I have an appropriate level of expertise to confirm that it is of an acceptable scientific standard.

# Peer review: Reviewer response for version 1

**DOI:** 10.21956/hrbopenres.14118.r27663

**Published:** 2020-07-29

**Authors:** Vicki L Gray

**Affiliations:** 1 Department of Physical Therapy and Rehabilitation Science, University of Maryland School of Medicine, Baltimore, MD, USA

This is a protocol for an umbrella review that will examine the effectiveness of non-pharmacological fall prevention interventions in people with Multiple Sclerosis, Parkinson’s Disease, and Stroke. The goal is to provide clinical recommendations that can span across these populations.

 In regards to the introduction portion of the protocol, the authors should discuss limitations related to the heterogeneity within conditions and across conditions, such as chronicity (acute stroke vs. chronic stroke), degree of impairment (ambulatory vs. non-ambulatory), and mechanisms of impaired performance. All of these may impact the ability to aggregate the interventions across conditions and within one condition.

 Overall, it is not clear whether there will be enough data for an umbrella review. Chien-Hung *et al*., (2019) ^1^ only found 15 reviews; and, their assessment included 8 reviews related to dementia/MCI/diabetes. The authors did not provide a clear explanation as to why they chose these populations. It would seem as if this area would benefit from a systematic review.

 There are also some methodological details not included in the manuscript, such as

- Who will be performing the search of the databases?
- Exclusion criteria – will this include systematic reviews without a search strategy?
- What type of data will be extraction for the outcome measures – means, confidence, or effect sizes? Effective sizes would be of more value.

Please clarify.

 As an example, the authors stated they would include systematic reviews with RCT along with other study designs. However, the comparison is usual or enhance care (Table 1). This would eliminate system reviews that include a single group design. Be more specific on the type of studies in the systematic reviews that will determine whether the study will be included or excluded.

 Many studies do not measure falls as a primary outcome measure. However, based on the Table 1 summary of outcomes - it seems that studies with falls as a primary outcome will be the only studies included. Clarify if it is a secondary measure; for example, to gait or balance that might be primary will it be included.

Is the study design appropriate for the research question?

Yes

Is the rationale for, and objectives of, the study clearly described?

Partly

Are sufficient details of the methods provided to allow replication by others?

Partly

Are the datasets clearly presented in a useable and accessible format?

Not applicable

Reviewer Expertise:

Balance and falls in stroke.

I confirm that I have read this submission and believe that I have an appropriate level of expertise to confirm that it is of an acceptable scientific standard, however I have significant reservations, as outlined above.

## References

[1] : Exercise Interventions for Individuals With Neurological Disorders: A Systematic Review of Systematic Reviews. *Am J Phys Med Rehabil*.98(10) : 10.1097/PHM.0000000000001247 921-930 10.1097/PHM.0000000000001247 31206360

# Response

**Published:** 2020-11-24

**Authors:** Nicola O'Malley

**Affiliations:** University of Limerick, Ireland

Many thanks for your time spent reviewing this protocol and for your constructive and insightful feedback and comments. We have reflected upon your feedback and made revisions to our manuscript in line with it. Please see below a detailed point by point response to all comments (reviewer's comments in **bold** and authors’ responses in black). Changes to the manuscript itself have been added in *italics*.

**Comment 1):** **In regards to the introduction portion of the protocol, the authors should discuss limitations related to the heterogeneity within conditions and across conditions, such as chronicity (acute stroke vs. chronic stroke), degree of impairment (ambulatory vs. non-ambulatory), and mechanisms of impaired performance. All of these may impact the ability to aggregate the interventions across conditions and within one condition.**

 Thank you for this comment. Reviewer 1 also sought some clarity regarding the feasibility of aggregating an intervention across conditions. However, we are off the belief that once the core elements underpinning the intervention remain constant, that interventions can be individualised and open to modifications based on the presenting participants. We have clarified our rationale for combining the three conditions in the introduction as follows:

*While the pathophysiology of stroke, PD and MS differs (3-6), there are similarities in the presenting impairments and falls risk factors across the three diagnoses. People with MS, PD and stroke share a number of physiological and psychosocial falls risk factors including impaired mobility, reduced balance, cognitive deficits, decreased strength, depression, fear of falling and reduced ability to perform activities of daily living (7-13), in addition to behavioural and environmental falls risk factors. Physiotherapists specialising in neurology or working in primary care usually manage individuals with each of these mixed neurological diseases in their practices and so, given the commonalities in these modifiable falls risk factors, it is likely that the subsequent goals of rehabilitation and treatment approaches used across diagnoses are also similar to reduce falls. This similarity in treatment approaches is reflected in research, where exercise with the aim of improving strength and balance appears to be the main component of many falls prevention interventions for people with PD, MS and stroke (14-16). Therefore, it is hypothesised that programmes for mixed neurological groups comprising of people with MS, PD and stroke are feasible. It is acknowledged that there will be some variation in clinical presentation between people with MS, PD and stroke; however, tailoring of a programme to an individual’s unique presentation is required for all interventions, independent of diagnosis. Many falls prevention interventions contain core elements underpinning the content and delivery of the programme, in addition to person-specific, individualised components; thus it is anticipated that this model could also be used to develop a programme for people with MS, PD and stroke that can be adapted based on individual falls risk assessments.*

 With respect to your specific concern regarding our ability to aggregate interventions due to this heterogeneity, we plan to have sub-analyses investigating differences in effect of interventions due to factors such as disease subtype, functional level, etc. The following has been added to the final paragraph of the introduction to clarify this:

*Additionally, this umbrella review will be the first of its kind to compare and contrast the effectiveness of interventions across diseases to facilitate the development of mixed neurological group interventions. These comparisons will consist of further sub-analyses to account for the heterogeneity both within and across the diagnoses of MS, PD and stroke, with respect to disease duration, functional ability and disease subtype.*

**Comment 2):** **Overall, it is not clear whether there will be enough data for an umbrella review. Chien-Hung et al., (2019)1 only found 15 reviews; and, their assessment included 8 reviews related to dementia/MCI/diabetes. The authors did not provide a clear explanation as to why they chose these populations. It would seem as if this area would benefit from a systematic review.**

 A primary difference between our review and the above mentioned is that we are looking at all non-pharmacological interventions whereas Chien-Hung et al. focused specifically on exercise-based interventions. We are confident that we will yield sufficient data for this umbrella review given our broader inclusion criteria with respect to interventions.

 The concept of taking a mixed-population approach to falls prevention seems relatively novel (as mentioned our scoping review only identified one RCT providing an intervention to people with different diagnoses). As a result, it seems intuitive to begin this process by trying to combine a small number of disorders. If this approach was found to be successful, then further consideration could be given to the idea of combining additional diseases. In terms of population, our primary reason for focusing on MS, PD and stroke are that these are three of the most prevalent neurological diseases in Ireland, and regularly present to clinics for falls rehabilitation.

*Neurological conditions are a leading cause of disability worldwide and, as a result, are associated with a large economic burden (1). The global expenditure for disability secondary to neurological disorders has increased substantially over the past few decades, and is expected to increase further in the coming decades due to rapid expansions in population ageing (1). In Ireland, three of the most prevalent neurological conditions are Multiple Sclerosis (MS), Parkinson’s Disease (PD) and stroke (2). Fall rates are high among people with these neurological diseases and are often associated with many negative consequences.*

**Comment 3):** **There are also some methodological details not included in the manuscript, such as Who will be performing the search of the databases?**

 Thank you, this has been clarified under ‘Search strategy’:

*The following electronic databases will be searched by one reviewer (NO’M) to identify potentially relevant reviews*

**Comment 4):** **Exclusion criteria – will this include systematic reviews without a search strategy?**

 We have clarified or inclusion/exclusion criteria for what constitutes a systematic review. As outlined in the updated section below, reviews that do not have an appropriate search strategy will be excluded as they will be deemed to be missing a key feature of accepted systematic review methodology:

*Any review that receives a ‘No’ response to any of the following will not be included (18, 19):*

*•           Were the inclusion criteria appropriate for the review question? (Item 2)*

*•           Was the search strategy appropriate? (Item 3)*

*•           Were the sources and resources used to search for the studies appropriate? (Item 4)*

*•           Were the criteria for appraising studies appropriate? (Item 5)*

*•           Were the methods used to combine studies appropriate? (Item 8)*

**

*Upon completion of this appraisal, literature reviews that do not include these key features of accepted systematic review methodology, outlined by JBI ^37^, will be excluded from this umbrella review. If necessary, the authors of the reviews will be contacted to clarify any unclear or missing details before the review is excluded.*

**Comment 5):** **What type of data will be extraction for the outcome measures – means, confidence, or effect sizes? Effective sizes would be of more value.**

**Please clarify.**

 Our aim is to present the data using one measure of effect (by converting all measures of effect to one standard measure), however, our ability to complete this will be largely influenced by the data that is available to us from the included reviews. We anticipate that there will be large variation between the outcomes presented in the reviews which would mean that our results will most likely be delivered through narrative synthesis with the presentation of quantitative results as able. Our plan for ‘Data synthesis and analysis’ has been updated as follows:

*We will endeavour to have a standardised approach to our results by converting the different estimates of effect that we extract to one common effect measure. However, these analyses will be contingent on several factors including access to raw data, whether the authors of the included systematic reviews performed meta-analyses and if the included systematic reviews have analysed the same falls outcomes. Given the anticipated heterogeneity in populations, outcomes and analyses, the findings of included reviews will likely be primarily summarised using a narrative synthesis with the quantitative tabulation of results as appropriate. The primary analyses for this umbrella review will be centred on type of neurological condition and type of intervention. Following this, cross-comparison of similarities and differences in the effect of different interventions between the three conditions will be performed. If the relevant data are presented in the included reviews, sub-analyses based on intervention dose, disease duration, functional ability and disease subtype will be completed.*

**Comment 6):** **As an example, the authors stated they would include systematic reviews with RCT along with other study designs. However, the comparison is usual or enhance care (Table 1). This would eliminate system reviews that include a single group design. Be more specific on the type of studies in the systematic reviews that will determine whether the study will be included or excluded.**

 Any systematic review in which the primary studies investigated the effects of a falls prevention intervention (regardless of the study design of the primary study) will be included. As RCTs are considered the gold-standard for determining effect, it is anticipated that the majority of included systematic reviews will have included RCTs, in that instance usual or enhanced care are the accepted comparators. This has been clarified in Table 1 under ‘Comparison’ and ‘Outcomes’.

**Comment 3.7):** **Many studies do not measure falls as a primary outcome measure. However, based on the Table 1 summary of outcomes - it seems that studies with falls as a primary outcome will be the only studies included. Clarify if it is a secondary measure; for example, to gait or balance that might be primary will it be included.**

 Falls measured as a primary or secondary outcome in systematic reviews will be included. This has been clarified in Table 1 under the heading ‘Outcomes’.

# Peer review: Reviewer response for version 1

**DOI:** 10.21956/hrbopenres.14118.r27665

**Published:** 2020-07-24

**Authors:** Feng Yang, Caroline Simpkins

**Affiliations:** 1 Department of Kinesiology and Health, Georgia State University, Atlanta, Georgia, USA

Falls are a common and serious health threat facing people with neurological diseases, such as multiple sclerosis, Parkinson’s disease, and stroke. The authors have made a strong argument for conducting an umbrella review to identify fall prevention interventions that are effective across neurological diseases. If one or more such interventions can be found, the clinical practice of fall prevention for people with neurological disorders could experience a paradigm shift away from the disease-specific approach, possibly leading to an efficient use of the limited resources for preventing falls.

 Overall, the manuscript is well written and the authors clearly explain the general approach and procedures of conducting this umbrella review. However, there are a few potential issues which may weaken the quality of this planned review project.

1. The authors should pre-determine the definition of a fall and what the exclusion/inclusion criteria are in terms of the circumstance/cause of falls. For example, some studies adopt falls with injuries as the outcome measure and some studies excluded falls due to environmental causes. Will injured falls and recurrent falls be considered separately?
2. It is unclear what the definitions of the three outcome measures are. It would be helpful for the readers if a detailed definition is given for each of the three outcome measurements: total number of falls, falls rate, and number of fallers.
3. We agree with the first reviewer that the quality, instead of the publication date, should be used as the standard to select the study in case of the presence of a complete overlap between reviews.
4. The authors stated that “If a review is an update of a previous review, the most recent update will be included and the older versions will be excluded.” It is unspecified if this rule only applies to updated reviews by the same author(s) or more generally to updated reviews on the same topic. Please clarify.
5. It is likely that the reviews analyzed the effect of interventions on falls using different summary measures or effect sizes (such as, rate ratio, risk ratio, or odds ratio). How will the authors handle this variability?
6. The authors mentioned that “Data will be extracted by one reviewer using a standardised data extraction form (NO’M). The extracted data will be verified by a second reviewer (AC/SC).” It is vague whether both reviewers will independently extract the data and then compare forms. Or is it proposed that only one reviewer will extract and then the second reviewer will agree/disagree based on those results? Clarification is needed.
7. Will adverse events and attrition rates be collected from each intervention and summarized? These two metrics could be critical factors when planning the deployment of fall prevention interventions to community or clinical settings.

Additionally, two minor and specific comments are listed below.

1. The PROSPERO registration number is not provided.
2. Please consider rephrasing the last sentence on page 5, beginning with “If sufficient reviews …”

Is the study design appropriate for the research question?

Yes

Is the rationale for, and objectives of, the study clearly described?

Yes

Are sufficient details of the methods provided to allow replication by others?

Partly

Are the datasets clearly presented in a useable and accessible format?

Not applicable

Reviewer Expertise:

Fall prevention and neurorehabilitation.

We confirm that we have read this submission and believe that we have an appropriate level of expertise to confirm that it is of an acceptable scientific standard, however we have significant reservations, as outlined above.

# Response

**Published:** 2020-11-24

**Authors:** Nicola O'Malley

**Affiliations:** University of Limerick, Ireland

Many thanks for your time spent reviewing this protocol and for your constructive and insightful feedback and comments. We have reflected upon your feedback and made revisions to our manuscript in line with it. Please see below a detailed point by point response to all comments (reviewer's comments in **bold** and authors’ responses in black). Changes to the manuscript itself have been added in *italics*.

**Comment 1):** **The authors should pre-determine the definition of a fall and what the exclusion/inclusion criteria are in terms of the circumstance/cause of falls. For example, some studies adopt falls with injuries as the outcome measure and some studies excluded falls due to environmental causes. Will injured falls and recurrent falls be considered separately?**

 Ideally, we would provide a pre-determined definition regarding what constitutes a fall. However, given that there is currently no consensus for the definition of a fall, and the variations in the definitions used in the literature, a pre-determined definition would restrict which systematic reviews would meet our inclusion criteria. Additionally, the use of a pre-determined definition has the potential to introduce bias into our review as we would only be including reviews that we believe meet the criteria for a fall. We plan to extract the definitions for a fall presented in the included reviews to provide context regarding the results for our readers.

 For this review, the occurrence of a fall event (regardless of how this fall was defined) had to be recorded to be considered a falls outcome. Both injurious falls and recurrent falls meet this criterion and as a result are considered primary outcomes, each of which will be analysed separately. This has been clarified in Table 1 under the subheading ‘Outcomes’.

****

**Comment 2):** **It is unclear what the definitions of the three outcome measures are. It would be helpful for the readers if a detailed definition is given for each of the three outcome measurements: total number of falls, falls rate, and number of fallers.**

****Thank you. A definition for each of the three outcomes have been added under the subheading ‘Quality of evidence’:

*1. Total number of falls - the number of falls recorded by participants throughout the study period*

*2. Falls rate - the number of falls per person per specific period of time, e.g. falls per person per year*

*3. Number of fallers - the proportion of participants classified as ‘fallers’ based on the criteria outlined by the researchers e.g. an individual who has one or more falls during the follow-up period (Note: it is anticipated that the classification for a ‘faller’ will differ between reviews (21), if this is the case the reviewers will present these differences and discuss the potential impact on the results).*

**Comment 3):** **We agree with the first reviewer that the quality, instead of the publication date, should be used as the standard to select the study in case of the presence of a complete overlap between reviews.**

 In accordance with this suggestion from both reviewers, the following update has been made to the manuscript under the heading ‘Overlap of primary studies’:

*In the presence of complete overlap between reviews, the highest quality review, as determined by the AMSTAR 2, will be included in data synthesis and analysis. In cases, where there is complete overlap and the reviews receive the same rating using the AMSTAR 2, then the most recently published review will be included.*

**Comment 4):** **The authors stated that “If a review is an update of a previous review, the most recent update will be included and the older versions will be excluded.” It is unspecified if this rule only applies to updated reviews by the same author(s) or more generally to updated reviews on the same topic. Please clarify.**

 This has been clarified under the subheading ‘Inclusion and exclusion criteria’:

*An update of a systematic review has changes pertaining to new data, new methods, or new analyses, however, the research question, objectives and inclusion criteria remain similar (17). This updated review may be conducted by the same authors as the previous review or the research team may comprise of new authors. In the case of new authors updating an existing review, they must clearly state that their review is an update and acknowledge the work of the authors on the previous edition (17).*

**Comment 5):** **It is likely that the reviews analyzed the effect of interventions on falls using different summary measures or effect sizes (such as, rate ratio, risk ratio, or odds ratio). How will the authors handle this variability?**

 If possible, our aim is to present the data using one measure of effect (by converting all measures of effect to one standard measure), however, our ability to complete this will be largely influenced by the data that is available to us from the included reviews. We anticipate that there will be large variation between the outcomes presented in the reviews which would mean that our results will most likely be delivered through narrative synthesis. Our plan for dealing with this variation between reviews has been updated under the section ‘Data synthesis and analysis’:

*We will endeavour to have a standardised approach to our results by converting the different estimates of effect that we extract to one common effect measure. However, these analyses will be contingent on several factors including access to raw data, whether the authors of the included systematic reviews performed meta-analyses and if the included systematic reviews have analysed the same falls outcomes. Given the anticipated heterogeneity in populations, outcomes and analyses, the findings of included reviews will likely be primarily summarised using a narrative synthesis with the quantitative tabulation of results as appropriate. The primary analyses for this umbrella review will be centred on type of neurological condition and type of intervention. Following this, cross-comparison of similarities and differences in the effect of different interventions between the three conditions will be performed. If the relevant data are presented in the included reviews, sub-analyses based on intervention dose, disease duration, functional ability and disease subtype will be completed.*

**Comment 6):** **The authors mentioned that “Data will be extracted by one reviewer using a standardised data extraction form (NO’M). The extracted data will be verified by a second reviewer (AC/SC).” It is vague whether both reviewers will independently extract the data and then compare forms. Or is it proposed that only one reviewer will extract and then the second reviewer will agree/disagree based on those results? Clarification is needed.**

 This has been clarified as follows under the section ‘Data extraction’:

*Data will be extracted by one reviewer (NO’M) using a standardised data extraction form. A second reviewer (LC) will then check the form to ensure that the extracted data are accurate.*

**Comment 7):** **Will adverse events and attrition rates be collected from each intervention and summarized? These two metrics could be critical factors when planning the deployment of fall prevention interventions to community or clinical settings.**

 We agree that adverse events and attrition rates could be critical factors in the planning and delivery of interventions and where available in included systematic reviews, both of these will be extracted as secondary outcomes. This has been clarified in Table 1 under the heading ‘Outcomes’.

**Comment 8):** **The PROSPERO registration number is not provided.**

 Thank you the PROSPERO registration number has been added under the subheading ‘Protocol and registration’:

*The protocol was registered with the International Prospective Register of Systematic Reviews, PROSPERO, CRD42020175409.*

**Comment 9):** **Please consider rephrasing the last sentence on page 5, beginning with “If sufficient reviews …”**

 This sentence has been rephrased as follows:

*Where possible, the sensitivity of the review findings will be considered in the context of its methodological quality, as determined by the AMSTAR-2, to examine the effects of synthesising reviews of varying quality. In the first instance, analyses will be completed using systematic reviews of any methodological quality that include all study designs followed by a second analysis using only systematic reviews with highest quality evidence (RCTs only). Comparisons between the two analyses will then be presented and discussed.*

# Peer review: Reviewer response for version 1

**DOI:** 10.21956/hrbopenres.14118.r27347

**Published:** 2020-04-29

**Authors:** Ylva Nilsagård

**Affiliations:** 1 Health Care Management, Faculty of Medicine and Health, Örebro University, Örebro, Sweden

This is a study protocol for an umbrella review - registered in PROPSPERO - that will include systematic reviews of randomised controlled trials and studies investigating the effects of non-pharmacological falls prevention interventions on falls outcomes. The PRISMA checklist was used to review the study protocol.

 The following suggestions may help improving the protocol further.

 There is a wide range of neurological diseases and I therefore suggest that the authors refrain from using this expression when addressing the population of interest and instead use MS; PD and stroke. I also suggest that the authors problematise lumping together stroke, MS and PD since the underlying mechanisms to the diseases differ and explain how an intervention UNS will be advantageous for a person-centered care. The authors may want to elaborate whether the rationale to find sufficient numbers and resources to run single group interventions could mean that less specified and thus less effective interventions will be recommended in the future, i.e., is the rational strong enough?

 Beforehand decision on inclusion/exclusion criteria regarding length/intensity; supervised/unsupervised and content (training; assistive devices or orthosis; environmental changes; behavioural) of the interventions investigated should be expressed. Provide a rational for the decisions.

 Please provide a more stringent and yet more detailed PICO. For example age, disease duration, disease symptoms, co-morbidity etc.

 Add the systematic review registration number in PROSPERO.

 Information on quality rating of primary studies included in the reviews will be extracted. It would strengthen the review if the ratings were checked by the authors, using a predetermined tool.

 Cut-offs for categorizing the AMSTAR scores into high, moderate, low or critically low quality should be provided.

 Please consider to include the review with the highest quality if there is an complete overlap instead of including the most recent review.

 I strongly suggest that subanalyses for disease; type of intervention and total amount of intervention are considered.

Is the study design appropriate for the research question?

Yes

Is the rationale for, and objectives of, the study clearly described?

Partly

Are sufficient details of the methods provided to allow replication by others?

Yes

Are the datasets clearly presented in a useable and accessible format?

Not applicable

Reviewer Expertise:

Falls and balance training in people with MS.

I confirm that I have read this submission and believe that I have an appropriate level of expertise to confirm that it is of an acceptable scientific standard, however I have significant reservations, as outlined above.

# Response

**Published:** 2020-11-24

**Authors:** Nicola O'Malley

**Affiliations:** University of Limerick, Ireland

Many thanks for your time spent reviewing this protocol and for your constructive and insightful feedback and comments. We have reflected upon your feedback and made revisions to our manuscript in line with it. Please see below a detailed point by point response to all comments (reviewer's comments in **bold** and authors’ responses in black). Changes to the manuscript itself have been added in *italics*.

**Comment 1):** **There is a wide range of neurological diseases and I therefore suggest that the authors refrain from using this expression when addressing the population of interest and instead use MS; PD and stroke.**

 Thanks, this has been updated accordingly throughout the manuscript.

**Comment 2):** **I also suggest that the authors problematise lumping together stroke, MS and PD since the underlying mechanisms to the diseases differ and explain how an intervention UNS will be advantageous for a person-centered care.** **The authors may want to elaborate whether the rationale to find sufficient numbers and resources to run single group interventions could mean that less specified and thus less effective interventions will be recommended in the future, i.e., is the rational strong enough?**

 Thank you for this thought-provoking comment. While we acknowledge that there are differences in the mechanisms underpinning each disease, we believe that the similarities in fall risk factors across the three conditions mean that subsequent treatment approaches will also be similar and as a result a programme for people with MS, stroke and PD is feasible. We have updated our introduction as follows to clarify our rationale for combining these three neurological conditions:

*While the pathophysiology of stroke, PD and MS differs (3-6), there are similarities in the presenting impairments and falls risk factors across the three diagnoses. People with MS, PD and stroke share a number of physiological and psychosocial falls risk factors including impaired mobility, reduced balance, cognitive deficits, decreased strength, depression, fear of falling and reduced ability to perform activities of daily living (7-13), in addition to behavioural and environmental falls risk factors. Physiotherapists specialising in neurology or working in primary care usually manage individuals with each of these mixed neurological diseases in their practices and so, given the commonalities in these modifiable falls risk factors, it is likely that the subsequent goals of rehabilitation and treatment approaches used across diagnoses are also similar to reduce falls. This similarity in treatment approaches is reflected in research, where exercise with the aim of improving strength and balance appears to be the main component of many falls prevention interventions for people with PD, MS and stroke (14-16). Therefore, it is hypothesised that programmes for mixed neurological groups comprising of people with MS, PD and stroke are feasible. It is acknowledged that there will be some variation in clinical presentation between people with MS, PD and stroke; however, tailoring of a programme to an individual’s unique presentation is required for all interventions, independent of diagnosis. Many falls prevention interventions contain core elements underpinning the content and delivery of the programme, in addition to person-specific, individualised components; thus it is anticipated that this model could also be used to develop a programme for people with MS, PD and stroke that can be adapted based on individual falls risk assessments.*

**Comment 3):** **Beforehand decision on inclusion/exclusion criteria regarding length/intensity; supervised/unsupervised and content (training; assistive devices or orthosis; environmental changes; behavioural) of the interventions investigated should be expressed. Provide a rational for the decisions.**

 The inclusion/exclusion criteria for interventions has been clarified in Table 1 under the heading ‘Intervention’.

**Comment 4):** **Please provide a more stringent and yet more detailed PICO. For example age, disease duration, disease symptoms, co-morbidity etc.**

 A more detailed PICO has been added to Table 1.

**Comment 5):** **Add the systematic review registration number in PROSPERO.**

 Thank you the PROSPERO registration number has been added under the subheading ‘Protocol and registration’.

*The protocol was registered with the International Prospective Register of Systematic Reviews, PROSPERO, CRD42020175409.*

**Comment 6):** **Information on quality rating of primary studies included in the reviews will be extracted. It would strengthen the review if the ratings were checked by the authors, using a predetermined tool.**

 We agree that the use of a predetermined tool to assess the quality rating of primary studies in included systematic reviews can strengthen the review. However, for this umbrella review our unit of analysis is the systematic review and consequently, we will not be performing a quality rating of the primary studies. As part of the JBI Critical Appraisal Checklist for Systematic Reviews and Research Syntheses, we will record and present whether systematic reviews completed critical appraisal by two or more reviewers independently. Additionally, as part of the AMSTAR 2 we will be recording if the authors of the systematic reviews used a satisfactory tool to assess for risk of bias.

**Comment 7):** **Cut-offs for categorizing the AMSTAR scores into high, moderate, low or critically low quality should be provided.**

 The cut-offs for the AMSTAR 2 have been added under the subheading ‘Methodological quality assessment:

*In line with recommendations (20), the following will be considered critical domains for the AMSTAR 2: *

- *Protocol registered before commencement of the review (item 2)*
- *Adequacy of the literature search (item 4)*
- *Justification for excluding individual studies (item 7)*
- *Risk of bias from individual studies being included in the review (item 9)*
- *Appropriateness of meta-analytical methods (item 11)*
- *Consideration of risk of bias when interpreting the results of the review (item 13)*
- *Assessment of presence and likely impact of publication bias (item 15)*

*The overall confidence in the results of a systematic review will be considered high if it has no or one non-critical weakness, moderate if more than one non-critical weakness is present, low if there is one critical flaw with or without non-critical weaknesses present, and critically low if there is more than one critical flaw with or without non-critical weaknesses (20).*

**Comment 8):** **Please consider to include the review with the highest quality if there is a complete overlap instead of including the most recent review.**

 Thank you for this suggestion, the following update has been made to the manuscript under the subheading ‘Overlap of primary studies’:

*In the presence of complete overlap between reviews, the highest quality review, as determined by the AMSTAR 2, will be included in data synthesis and analysis. In cases, where there is complete overlap and the reviews receive the same rating using the AMSTAR 2, then the most recently published review will be included.*

**Comment 9):** **I strongly suggest that sub-analyses for disease; type of intervention and total amount of intervention are considered.**

 Thank you for this suggestion. As the aim of this umbrella review is to determine the effectiveness of non-pharmacological falls prevention interventions for people with MS, PD and stroke, our primary analyses will be based on disease and type of intervention. Our ability to perform a sub-analyses based on intervention dose will be determined by the data available to us in the included reviews, however, we will consider this as an option if able. Once we have the findings for each condition and type of intervention we will then compare our findings across the three conditions to identify similarities and differences in effect. The section ‘Data synthesis and analysis’ has been updated as follows:

*The primary analyses for this umbrella review will be centred on type of neurological condition and type of intervention. Following this, a cross-comparison of similarities and differences in the effect of different interventions between the three conditions will be performed. If the relevant data are presented in the included reviews, sub-analyses based on intervention dose, disease duration, functional ability and disease subtype will also be completed.*
